# Navigating the Second Victim Experience in Gastrointestinal Endoscopy and Colonoscopy

**DOI:** 10.1002/jgh3.70215

**Published:** 2025-07-01

**Authors:** Hareesha Rishab Bharadwaj, Syed Hasham Ali, Aditya Gaur, Muhtasim Fuad, Matan Bone, Khabab Abbasher Hussien Mohamed Ahmed, Dushyant Singh Dahiya

**Affiliations:** ^1^ Faculty of Biology Medicine and Health The University of Manchester Manchester UK; ^2^ Dow University of Health Sciences Karachi Pakistan; ^3^ Yeovil District Hospital, Somerset NHS Foundation Trust, Higher Kingston Yeovil UK; ^4^ Salford Royal Hospital, Northern Care Alliance NHS Foundation Trust Salford UK; ^5^ University of Khartoum Khartoum Sudan; ^6^ Division of Gastroenterology Hepatology and Nutrition The University of Kansas Medical School Kansas USA

## 1 Introduction

Gastrointestinal endoscopy is a cornerstone of modern gastroenterological practice, offering both diagnostic and therapeutic benefits with a commendably high safety profile. Nevertheless, despite its minimally invasive nature, endoscopic procedures are not without risk. Significant complications and adverse events (AEs) can occur. Certain studies reveal that up to 85% of colonoscopists experience AEs [1]. In the UK, for instance, AE rates range from 2.8 per 1000 colonoscopies in screening populations to 5 per 1000 in symptomatic patients—figures that align closely with international data [2]. Within the NHS Bowel Cancer Screening Programme, which evaluated 263 129 colonoscopies between 2006 and 2014, the perforation rate was reported at a low 0.06% [3]. Importantly, complication risks are notably higher in older adults; a JAMA study demonstrated significantly elevated 30‐day post‐colonoscopy complication rates in individuals aged ≥ 75 compared to those aged 50–74. Diagnostic colonoscopies also carry a higher risk (0.1%) relative to screening procedures (0.04%) [2, 3, 4]. Colonoscopy‐related 30‐day mortality remains low, estimated between 1 in 100 000 and 1 in 10 000 procedures [5, 6].

While the medical management of complications such as perforation, bleeding, pancreatitis, and cholangitis is well established—supported by evidence‐based protocols—far less attention has been given to the broader implications for endoscopists themselves. The emotional, professional, and medico‐legal consequences of AEs can be substantial, directly impacting clinicians' well‐being, confidence, and quality of care provided [7].

Injuries resulting from medical management, rather than the underlying disease, can arise from a spectrum of circumstances ranging from preventable errors to unavoidable complications [1]. Although attention is often directed towards patient outcomes, issues such as miscommunication, insufficient informed consent, and lack of systemic support frequently intensify the endoscopist's burden [1, 8]. This underscores the pressing need for comprehensive strategies that not only focus on preventing complications but also provide meaningful support for clinicians managing their aftermath. Notably, some studies suggest that over half of AEs may be preventable with improved systems and training. Still, even when unavoidable, such events can significantly undermine an endoscopist's confidence and professional standing [1].

The emotional toll is particularly profound for trainees, who may lack the experience, mentorship, and structured guidance to manage complications effectively. Current training programs often inadequately address non‐technical aspects of AE management—including disclosure, apology, and patient communication—leaving clinicians to confront these challenges without sufficient preparation or support. The absence of formal mechanisms for peer support, structured debriefing, and institutional backing can further exacerbate feelings of isolation and burnout [1, 2, 5].

Recognizing this critical gap, this article advocates for a holistic approach to endoscopy‐related complication management—one that integrates technical proficiency with emotional resilience, robust peer support systems, and institutional frameworks designed to sustain both the clinician and the quality of care.

## 2 Effects of Adverse Events on Endoscopists

AEs in gastrointestinal endoscopy can have profound effects on endoscopists, impacting their emotional, physical, social, and professional well‐being. These events, particularly when they result in patient harm, often trigger intense psychological responses, including feelings of guilt, anxiety, and sadness. The emotional burden is typically exacerbated by the endoscopist's empathy for the patient and the perception of failing to meet procedural safety standards. Studies have shown that up to 40.4% of colonoscopists who experience complications such as perforations undergo significant psychological distress, including self‐doubt and diminished professional self‐efficacy. The emotional impact is further intensified when complications are not immediately identified, leading to delayed treatment and longer hospital stays for the patient, thereby prolonging the psychological strain on the clinician. This erosion of self‐confidence can lead to burnout, which is characterized by emotional exhaustion, depersonalization, and reduced professional efficacy. Persistent psychological distress, often manifesting as self‐doubt and rumination, can be linked to the extent of harm caused and the clinician's relationship with the patient. Furthermore, AEs can disrupt team dynamics, leading to miscommunication, mistrust, and misplaced blame, which further undermine morale and cohesion within clinical teams [8, 9, 10].

Endoscopists who experience AEs are often referred to as “second victims.” This term describes the significant psychological stress that they endure following such events. This phenomenon is particularly prevalent among colonoscopists, with high rates of embarrassment (95.5%), fear of future incidents (75%), and remorse (44%) being reported after complications such as colonic perforations [1]. Initial reactions to AEs commonly include anxiety, guilt, and social withdrawal, but these can progress over time into chronic issues, such as depression and insomnia [8, 9, 10]. Addressing the needs of these “second victims” is crucial to fostering clinician resilience, maintaining positive team dynamics, and ensuring the provision of safe, high‐quality care.

Empirical literature indicates that the second victim phenomenon profoundly affects all grades of the gastrointestinal workforce, including attendings, junior residents, and endoscopy nurses, though the nature and intensity of their responses vary [7, 11]. Junior residents often experience pronounced anxiety, guilt, and prolonged self‐doubt, driven by concerns about clinical competence and future career prospects. In contrast, nurses frequently report moral distress and emotional exhaustion, particularly when required to continue providing patient care immediately following an adverse event. Seasoned attendings are not immune; they too can be deeply affected, often internalizing blame and ruminating over the clinical decisions that preceded the incident. One senior endoscopist described a colonoscopy‐related perforation that “stayed with me for days, weeks, and maybe months,” as he continually questioned whether he had missed warning signs and felt he had “failed” the patient. Recovery trajectories also differ by experience level. Less experienced clinicians, including junior doctors and younger nurses, typically require more time to recover, with evidence suggesting they struggle more to process and reconcile the emotional aftermath of adverse events [12, 13, 14].

In addition to the emotional toll, the medico‐legal consequences of AEs can significantly exacerbate the psychological burden experienced by endoscopists. Serious complications—such as colonoscopic perforation or post‐polypectomy bleeding—frequently lead to litigation, carrying profound financial, professional, and reputational implications. One study found that 91% of lawsuits related to colon perforation were ruled in favor of the plaintiff, with average compensation reaching $47917.83. In Korea, the median legal compensation for perforation was reported as $9335—approximately 130 times the cost of a standard colonoscopy [8]. In Western healthcare systems like those in the UK and US, legal settlements for severe injuries can escalate into millions [1].

Beyond individual consequences, the second victim phenomenon also imposes substantial economic costs on healthcare systems. Direct costs include litigation, compensation, and additional clinical interventions following AEs. Indirect costs arise from healthcare provider burnout, absenteeism, staff turnover, and the adoption of defensive medical practices. For example, physician burnout alone is estimated to cost the U.S. healthcare system approximately $4.6 billion annually [13, 15]. In a Singaporean study, 31% of nurses involved in adverse events reported considering leaving their job, and 9.3% reported absenteeism as a consequence [16]. Defensive medical behaviors—such as unnecessary investigations or avoiding high‐risk procedures—can further inflate costs and paradoxically reduce the overall quality and efficiency of care [12].

Moreover, the institutional impact of serious AEs can be far‐reaching. Adverse events may damage an organization's reputation, erode staff morale, and disrupt team dynamics—phenomena collectively described as the “third victim” effect [17]. The fear of litigation and reputational harm can also deter endoscopists from seeking emotional or professional support, thereby compounding their psychological distress. Instead of openly discussing the incident, some may withdraw from colleagues or suppress their emotions, further deepening their sense of isolation and exacerbating the second victim experience [12, 15, 17].

The disconnect between the perceptions of clinicians and patients regarding the necessity of compensation following an AE further complicates the emotional and legal challenges. Patients may view complications as errors that require compensation, whereas endoscopists, who view these events as inherent risks of the procedure, may not always recognize the need for compensation or an apology. This gap in expectations can intensify both the legal and emotional difficulties, underscoring the importance of effective communication—particularly thorough informed consent and patient education—in bridging the divide between patient expectations and clinician perceptions [9, 18].

Peer support is widely recognized as a critical resource for endoscopists dealing with the psychological aftermath of AEs. However, endoscopists often hesitate to seek such support due to fears of judgment or stigma, which can lead to reliance on maladaptive coping mechanisms such as emotional withdrawal and avoidance [13]. These behaviors only serve to exacerbate the psychological impact of AEs, and over time, unresolved emotional distress may contribute to burnout.

The psychological toll of AEs also manifests as physical symptoms, with affected endoscopists commonly reporting sleep disturbances, fatigue, and impaired concentration [12, 19]. Prolonged stress can lead to systemic health issues, such as hypertension and cardiovascular problems. Chronic stress, compounded by sleep deprivation, further exacerbates these physical symptoms, amplifying the clinician's overall burden. In some cases, unhealthy coping strategies, such as reduced physical activity or increased reliance on stimulants, are adopted, which further undermine physical health [4, 5, 6, 9].

AEs also have a significant impact on the personal lives of endoscopists. Feelings of inadequacy and failure can lead to social withdrawal, reducing opportunities for emotional support and increasing isolation. Emotional detachment, irritability, and preoccupation with the AE can place strain on personal relationships with family and friends. Cultural norms in medicine that discourage expressions of vulnerability can delay recovery and perpetuate unresolved emotional distress [13]. Additionally, concerns about medico‐legal implications and potential damage to professional reputation may prevent clinicians from seeking necessary psychological or peer support [1, 13, 15].

The professional consequences of AEs are equally significant. Cumulative psychological distress can erode job satisfaction, induce persistent self‐doubt, and, in some cases, cause clinicians to consider leaving the profession [13, 15]. Defensive medical practices, such as avoiding high‐risk procedures or over‐relying on diagnostic testing, are often adopted as risk mitigation strategies. While these behaviors may offer temporary reassurance, they typically reduce procedural efficiency and compromise patient outcomes. Nevertheless, some endoscopists are able to transform AEs into opportunities for growth by engaging in reflective practice and incorporating feedback [8]. These strategies can enable clinicians to refine their technical skills, enhance clinical decision‐making, and strengthen their commitment to patient‐centered care, showcasing their adaptability [13, 15].

The effects of adverse events on endoscopists are multifaceted, affecting their emotional, physical, and professional well‐being. A comprehensive approach to supporting these clinicians, through peer support, professional development, and effective communication, is vital to mitigating the impact of these events and promoting resilience. Addressing the psychological and social challenges associated with AEs can help maintain the well‐being of endoscopists, improve team dynamics, and ultimately ensure the continued provision of high‐quality patient care.

## 3 Discussion and Future Prospects for Navigating the Second Victim Phenomenon

Gastroenterologists are trained to work in high‐pressure situations, wherein the emotional and psychological toll of their profession can often be overlooked. Immediate access to specialized psychological support tailored to these unique challenges is essential. Establishing dedicated support programs within gastroenterology departments can help address the emotional toll of complications, patient outcomes, and other unique multifaceted challenges. Structured mental health support has been shown to reduce burnout, improve job satisfaction, and mitigate stress among healthcare professionals [12].

Creating a supportive environment where endoscopists feel comfortable seeking help without stigma is crucial. Encouraging open dialogue among colleagues and implementing structured debriefings after challenging cases can help foster resilience, improve team dynamics, and enhance patient safety. These reflective practices not only contribute to improved procedural outcomes but also support emotional well‐being, offering opportunities to learn from AEs to improve clinical practice [12, 13].

Given the inherent risks associated with optical colonoscopy, alternative diagnostic modalities—such as CT colonography (CTC), colon capsule endoscopy (CCE), and abdominal ultrasound—are being increasingly explored to reduce procedural complications and, by extension, mitigate the second victim phenomenon among endoscopists. CTC offers a non‐invasive imaging option with no sedation required and a perforation rate of just 0.02%–0.04%, markedly lower than colonoscopy. Most CTC‐related perforations are asymptomatic and do not require surgery, with surgical intervention needed in only 0.008% of cases across large‐scale meta‐analyses [20]. Although a follow‐up colonoscopy is still required for therapeutic interventions, CTC provides a valuable alternative, particularly for frail or high‐risk patients, and helps alleviate clinician anxiety following prior complications [21].

Similarly, CCE allows mucosal visualization without instrument insertion or sedation and has an excellent safety profile. No significant adverse events have been reported in major trials, and capsule retention is rare [10]. While its sensitivity is slightly lower for small polyps and it lacks therapeutic capabilities, CCE is especially beneficial for patients who are unable or unwilling to undergo colonoscopy. Abdominal ultrasound, although limited in detecting intraluminal lesions, remains valuable in triage and IBD assessment. It carries virtually zero risk, and its judicious use may reduce unnecessary endoscopies, contributing to a broader culture of safety [16]. From a psychological perspective, these modalities reduce the likelihood of provider‐inflicted harm and can serve as “step‐down” options for clinicians recovering from the trauma of previous adverse events, thereby restoring confidence and reducing anticipatory stress [15, 17]. Though not replacements for therapeutic endoscopy, their strategic integration into clinical pathways may both enhance patient safety and protect provider well‐being.

Telepsychiatry has become an increasingly valuable asset in addressing the mental health challenges faced by endoscopists. Implementing telehealth interventions like virtual counseling or well‐being programs provides endoscopists with immediate access to mental health support, especially in environments where in‐person resources may be limited [16, 22]. Furthermore, telepsychiatry has proven effective in reducing the stigma associated with mental health issues, encouraging clinicians to engage in self‐care strategies such as mindfulness training and stress reduction techniques [16, 20, 23]. Virtual well‐being programs, specifically designed for endoscopists, can enhance self‐reflection and stress management. These programs are vital for preventing burnout and supporting long‐term mental health without disrupting clinical duties [24]. Telemedicine's integration into post‐procedure debriefings allows for ongoing psychological support, helping endoscopists manage challenging cases while safeguarding their mental health [16, 22].

Training for endoscopists must go beyond technical proficiency to include psychological resilience and coping strategies. Emotional and psychological demands play a significant role in the well‐being of practitioners, and thus, resilience training should be integrated into training programs. Future training curricula should equip trainees with the tools to manage emotional and professional stress, communicate effectively in challenging situations, and access psychological support when needed. By prioritizing mental health alongside clinical skills, we can help endoscopists navigate the emotional and professional challenges of their roles, ensuring sustainable careers in this demanding field [15, 17, 24].

## Ethics Statement

The authors have nothing to report.

## Conflicts of Interest

The authors declare no conflicts of interest.

## Data Availability

Data availability is not applicable to this article as no new data were created or analyzed in this study.
